# A Hierarchical Motion Planning Method for Mobile Manipulator

**DOI:** 10.3390/s23156952

**Published:** 2023-08-04

**Authors:** Hanlin Chen, Xizhe Zang, Yubin Liu, Xuehe Zhang, Jie Zhao

**Affiliations:** State Key Laboratory of Robotics and Systems, Harbin Institute of Technology, Harbin 150001, China; zangxizhe@hit.edu.cn (X.Z.); zhangxuehe@hit.edu.cn (X.Z.); jzhao@hit.edu.cn (J.Z.)

**Keywords:** mobile manipulator, motion planning, hierarchical planner

## Abstract

This paper focuses on motion planning for mobile manipulators, which includes planning for both the mobile base and the manipulator. A hierarchical motion planner is proposed that allows the manipulator to change its configuration autonomously in real time as needed. The planner has two levels: global planning for the mobile base in two dimensions and local planning for both the mobile base and the manipulator in three dimensions. The planner first generates a path for the mobile base using an optimized A* algorithm. As the mobile base moves along the path with the manipulator configuration unchanged, potential collisions between the manipulator and the environment are checked using the environment data obtained from the on-board sensors. If the current manipulator configuration is in a potential collision, a new manipulator configuration is searched. A sampling-based heuristic algorithm is used to effectively find a collision-free configuration for the manipulator. The experimental results in simulation environments proved that our heuristic sampling-based algorithm outperforms the conservative random sampling-based method in terms of computation time, percentage of successful attempts, and the quality of the generated configuration. Compared with traditional methods, our motion planning method could deal with 3D obstacles, avoid large memory requirements, and does not require a long time to generate a global plan.

## 1. Introduction

A mobile manipulator consists of a mobile base and one (or more) traditional manipulators [1,2]. It integrates the advantages of both mobile robots and fixed-base manipulators. With the increased degrees of freedom provided by the mobile base, mobile manipulators could expand their workspace to a large area while maintaining the ability of dexterous manipulation of the manipulator.

At an early stage, most of the work focused on the problem of motion coordination between the mobile base and the manipulator [3,4,5]. Motion planning for mobile manipulators in this paper could be defined as determining a collision-free path from a given start configuration to a desired goal configuration. There are roughly two types of approaches to the problem. One of them is to consider the mobile manipulator as two subsystems and plan separately for the mobile base and the manipulator, the other one is to consider the whole mobile manipulator as a single system with high degrees of freedom. Separate planning for the mobile base and manipulator is relatively straightforward, various planning algorithms can be used for the mobile base [6,7] and the manipulator. Castaman et al. proposed an algorithm called Receding Horizon Task and Motion Planning (RH-TAMP), which used RRT-Connect for the manipulator planning and used Djikstra’s algorithm for the mobile base planning [8]. Saoji et al. used RRT-Connect to plan for both the mobile base and the manipulator while the planning was carried out separately [9]. Rastegarpanah et al. used the A* algorithm to plan for the mobile base and the RRT algorithm to plan for the manipulator [10]. Obviously, this type of method has a limitation, the manipulator could collide with the environment while the mobile base moves along the generated path because the effect of the environment on the manipulator along the path is ignored. Considering the influence of the environment, it seems to be a better choice to consider the whole mobile manipulator as a combined system with high DOF. In the simplest case, a very conservative approach is to plan for a 2D projected footprint of the mobile manipulator in a projected 2D representation of the world from the start base pose to the goal base pose while the manipulator only maintains a safe configuration [11]. The limitation of this type of methods is that the manipulator may extend the footprint of the robot which makes it difficult to find an available path since a footprint may be in collision with the 2D projected map while the mobile manipulator is collision-free in the 3D environment. Some researchers [12,13] considered motion planning as a trajectory optimization problem and proposed STOMP and CHOMP, where the goal is to find suitable trajectories that minimize a given cost function without violating the given constraints. Some researchers use sampling-based planners such as RRT [14], PRM [15], their variants RRT-Connect [16], RRT* [17], Bidirectional RRT* [18], and Informed RRT* [19] for mobile manipulator motion planning due to their ability to plan in high-dimensional space. The sampling-based planners could find solutions, but the path quality could be low due to the randomness associated with sampling-based algorithms even after post-processing. These methods [20,21] require a full 3D map of the environment, which is computationally expensive, and the map needs to be updated when the environment changes. Similarly, method [22] uses the potential field function to find a path through the free space represented as a swept volume, which is even more difficult to compute explicitly. 

In most cases, the mobile manipulator does not need to change the configuration of its manipulator continuously while the mobile base moves, the manipulator only needs to change its configuration at certain base poses. With this idea of adaptive dimensionality, some methods have been proposed that consider the behavioral difference between the mobile base and the manipulator with better performance. Gochev et al. [23] have proposed a method for mobile manipulator motion planning where planning in high dimension is performed only when planning in low dimension could not find an available path. Pilania and Gupta proposed the Hierarchical and Adaptive Mobile Manipulator Planner (HAMP) [24]. The planner first plans the mobile base path using PRM and then checks the collision between the manipulator and the environment for each edge along the path, if a collision is found, the manipulator changes its configuration by building a manipulator PRM. These methods ease the workload of planning in high-dimensional spaces but still need to plan in high dimension and still suffer from the long time consumed in computing a feasible path. Moreover, to generate a global plan for a mobile manipulator, a complete 3D map of the environment must be given to the planner first. Another planner with adaptive planning dimension called Optimized Hierarchical Mobile Manipulator Planner (OHMP) was proposed by Li et al. [25]. A path for mobile base was first planned with PRM, then at each mobile base node, if the manipulator was in collision with the environment, the manipulator should change to a safe configuration. The collision checking for the manipulator was performed using a 2D projection of the manipulator on the ground, which is much faster than checking in 3D maps. The disadvantage of this method is that if the manipulator is not a planar manipulator, the collision checking on 2D maps could not ensure that the manipulator would not collide with the 3D environment. 

Our approach adopts the idea of adaptive dimensionality. Planning for manipulator motion is conducted only when a potential collision between manipulator and environment is found. To avoid expensive computation of planning in high-dimension, our approach does not plan a global path for both the mobile base and the manipulator with a full 3D map of the environment, as some methods do [22,23]. In our approach, a full 2D map of the environment is needed to generate a global path for the mobile base, then a potential collision checking between the manipulator and the environment is performed while the mobile base is moving, if a potential collision is found, a safe configuration for the manipulator would be found. Like OHMP [24], our approach uses the 2D projection of the manipulator for potential collision checking and safe configuration generation, the difference is that our approach projects the manipulator onto the front plane of the mobile manipulator instead of the ground, which is suitable for manipulators that are not planar manipulators. Compared with similar methods [22,23,24], our method does not use a full 3D map of the environment and does not suffer from long time-consuming planning in high dimensions, but has better performance and efficiency in crossing door-type scenes.

In this paper, we divide the motion planning for mobile manipulators into two layers. In the first layer, the path planning for the mobile base is performed, which mainly focused on moving the robot in a large area. The second layer mainly focuses on motion planning for the manipulator to avoid collision. Our contribution is to propose hierarchical motion planning for the mobile manipulator and the sampling-based heuristic manipulator planning.

The rest of this paper is organized as follows. Section 2 defines the general problem and the parameters used in the paper. Section 3 describes the motion planning algorithm. Simulation results and discussions are given in Section 4. Finally, the conclusions are given in Section 5. 

## 2. Problem Formulation

The parameters used in this paper are defined below, and then the problem is formally formulated.

- $C_{bm}$, the configuration space of the mobile manipulator
- $C_{b}$, the configuration space of the mobile base
- $C_{m}$, the configuration space of the manipulator
- $C_{b_{free}}$, the set of all collision-free base poses
- $C_{b_{obs}}$, the set of all poses resulting in the collision with obstacles
- $C_{m_{free}}$, for a given base pose, $C_{m_{free}}$ is the set of all collision-free manipulator configurations
- $C_{m_{obs}}$, for a given base pose, $C_{m_{obs}}$ is the set of all configurations that are in collision with obstacles
- $q_{i}^{b}=[x,y,\varphi ]$, the pose of mobile base
- $q_{i}^{m}=[\theta_{1},\theta_{2},\cdots ,\theta_{d}]$, the configuration of the manipulator of d DOF
- $q_{i}=(q_{i}^{b},q_{i}^{m})$, the $i^{th}$ configuration of the mobile manipulator
- $q_{s}=(q_{s}^{b},q_{s}^{m})$, the starting configuration of the mobile manipulator
- $q_{g}=(q_{g}^{b},q_{g}^{m})$, the goal configuration of the mobile manipulator
- $Q=\{q_{s}^{b},q_{1}^{b},\cdots q_{n}^{b},q_{g}^{b}\}$, the path of the mobile base

For our motion planning method, the 2D environment of the mobile base is assumed to be given or acquired by previous sensing. The environment information in front of the robot is captured in real time by a depth camera mounted on the mobile manipulator. Given the 2D map of the environment, the environment information in front of the robot, the start configuration $q_{s}=(q_{s}^{b},q_{s}^{m})\in C_{bm}$, and the goal configuration $q_{g}=(q_{g}^{b},q_{g}^{m})\in C_{bm}$ of the mobile manipulator, the goal of our method is to find a collision-free path consisting of a set of mobile manipulator configuration $q_{i}=(q_{i}^{b},q_{i}^{m})\in C_{bm}$ for the mobile manipulator. The path in our method can be expressed as

(1) $$\Pi^{bm}=\{(q_{s}^{b},\pi_{s}^{m}),(q_{1}^{b},\pi_{1}^{m}),\cdots ,(q_{n}^{b},\pi_{n}^{m}),(q_{g}^{b},\pi_{g}^{m})\}.$$

Such a specific mobile manipulator path is called an H-path in HAMP [24]. An H-path consists of a set of mobile base poses $q_{i}^{b}=(x,y,\varphi )\in C_{b_{free}}$, and for each base pose, there is a corresponding manipulator reconfiguration path $\pi_{j}^{m}$. Each manipulator path $\pi_{j}^{m}$ is a set of manipulator configurations. Although the path expression used here is the same as that used in HAMP, the planning method is completely different. In HAMP, the entire path is generated at once, but in our method, the path is generated gradually, which is finally determined when the mobile manipulator arrives at the target. It is worth noting that, in most cases, the element in $\Pi^{bm}$, $\left(q_{s}^{b},\pi_{s}^{m}\right)$ can be simplified as $\left(q_{i}^{b},q_{i}^{m}\right)$, since the manipulator does not need to change its configuration. Only when a potential collision between the manipulator and the environment is found, a safe configuration of the manipulator would be sought, and a reconfiguration path would need to be generated. In our method, a collision-free global path *Q* consisting of a set of base configurations $q_{i}^{b}=(x,y,\varphi )\in C_{b_{free}}$ is first planned for the mobile base, at which time the entire path can be expressed as $\Pi^{bm}=\{(q_{s}^{b},q_{s}^{m}),(q_{1}^{b},q_{s}^{m}),\cdots ,(q_{n}^{b},q_{s}^{m}),(q_{g}^{b},q_{s}^{m})\}$. Second, while the mobile base moves along the path, collision checking for the manipulator is executed frequently, when a potential collision is found at $q_{i}^{b}$, a new collision-free configuration of the manipulator $q_{i}^{m}=(\theta_{1},\theta_{2},\theta_{3},\theta_{4},\theta_{5},\theta_{6},\theta_{7})\in C_{m_{free}}$ would be found, then a path $\pi_{i}^{m}$ for manipulator reconfiguration would be generated, and the element in the path $(q_{i}^{b},q_{i}^{m})\in \Pi^{bm}$ is updated to $\left(q_{i}^{b},\pi_{i}^{m}\right)$. There are two steps of mobile manipulator movement from $\left(q_{i}^{b},\pi_{i}^{m}\right)$ to $\left(q_{j}^{b},q_{j}^{m}\right)$; at $q_{i}^{b}$, the manipulator moves along $\pi_{i}^{m}$ to change to the last configuration $q_{j}^{m}$ in $\pi_{i}^{m}$, then the mobile base moves from $q_{i}^{b}$ to $q_{j}^{b}$ with the new manipulator configuration. Finally, when the mobile manipulator arrives at the target configuration, the entire H-path $\Pi^{bm}$ is fully incrementally determined.

## 3. Methods

In this section, we will discuss the overall motion planning problem for mobile manipulators.

Global planning of a mobile manipulator directly in a 3D environment requires not only a complete 3D map but also a large amount of memory and a long computation time. In most cases, the manipulator does not need to continuously change its configuration while the mobile base is moving. Considering that the mobile base and the manipulator have different motion characteristics, in the movement from one initial configuration to another target configuration, the long-range movement of the mobile base is the main action that the manipulator needs to cooperate with to avoid collision with the environment. Therefore, we adopt the idea of adaptive dimensionality and perform high-dimensional planning for the manipulator only when necessary and propose a hierarchical motion planning approach that performs global planning for the mobile base at the global planning level and adjusts the manipulator’s configuration as needed while controlling the mobile base motion at the local planning level. At the local planning level, to improve computational efficiency, we use a planar projection in front of the mobile manipulator perpendicular to the ground to detect potential collisions between the manipulator and the environment and determine whether the manipulator needs reconfiguration, based on which we propose a sampling-based heuristic to efficiently generate a collision-free safe configuration. Since this collision detection method and configuration generation method can only ensure that the robot does not collide with its environment directly in front of it, and the robot needs to ensure a straight line forward, a local planner very similar to FTC [26] is used in mobile base control to separate the rotational motion from the straight-line motion to ensure that it does not collide with the environment while moving forward. At the global planning level, an optimized A* planner is used to perform global path planning for the mobile base in order to facilitate the FTC planner to track the path, reducing the curves and simplifying the path.

**Figure 1:**
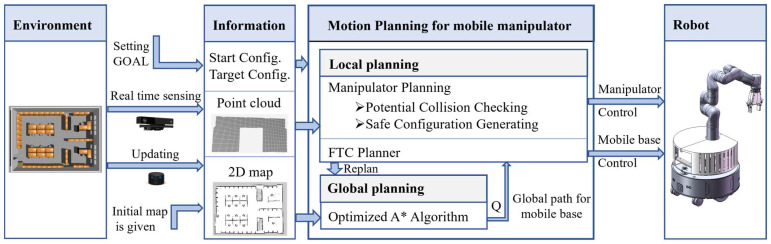
Hierarchical motion planning for mobile manipulator.

The structure of our Hierarchical Motion Planning for mobile manipulators is shown in Figure 1. The planner is divided into two levels. At the global planning level, an optimized A* algorithm is implemented to generate a global path for the mobile base assuming that the 2D map of the environment is known or acquired by previous sensing with a lidar. At the level of local planning, a modified FTC planner is used here to control the mobile base to move precisely along the global path, as for the manipulator, a potential collision checking is performed continuously with environment information gathered with sensors onboard, a new collision-free configuration for the manipulator would be searched and a reconfiguration path would be generated if a potential collision is found. Then the control signal for both the mobile base and the manipulator is calculated and sent to the robot so that the robot can continue to move to the target configuration. If local planning fails because there is no available path for the manipulator or the path of the mobile base is blocked, global planning would be called to re-plan a new global path with an updated 2D map. 

We now describe our hierarchical motion planning (HMP) algorithm in detail explained in Algorithm 1.

In the first stage, a routine PLANBASEPATH() is called to plan an initial global path for the mobile base using an optimized A* algorithm, the pseudocode for which is shown in Algorithm 2. The path planning for the mobile base is carried out on a 2D grid map. The 2D map can be constructed in advance using the gmapping algorithm [27] and the map is considered as the robot’s prior knowledge. Given the start and target configuration of the mobile base, an initial path of the mobile base is searched by A* algorithms [28]. The mobile base is simplified as a circle with a flattened radius to avoid collision with obstacles. 

A cutting method is used here to optimize the path generated by A* algorithm (Lines 3–17, Algorithm 2). The main idea of this method is to reduce the number of curves in the path and make it easier for the mobile base to move along. The path $Q_{origin}$ generated by A* algorithm has *n* elements $q_{i}^{b}\in C_{b_{free}}$, the first element is $q_{s}^{b}$ and the last element is $q_{g}^{b}$. The initial values of *q_prune_* and *q_target_* are $q_{s}^{b}$ and $q_{g}^{b}$. The algorithm has two loops. In the inner loop, for each iteration, try to connect *q_prune_* and *q_target_* with a line, if the line is not in collision with environment obstacles, those $q_{i}^{b}$ between them could be removed and the path is simplified, otherwise set *q_target_* as the *q*_*target*-1_ until *q_target_* is next to *q_prune_*. In the outer loop, set *q_prune_* as *q_target_* and set *q_target_* as *q_end_*, the algorithm goes into the inner loop until *q_prune_* is $q_{g}^{b}$. If there is no available path planned, routine PLANBASEPATH() returns failure.

**Figure 2:**
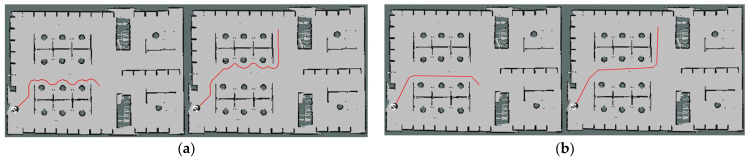
Global path comparison. (**a**) Path planned by A* algorithm; (**b**) Optimized path planned by optimized A* algorithm.

As shown in Figure 2, the two paths in Figure 2a are the original results generated by A* algorithm, and the two paths in Figure 2b are the optimized paths. It is obvious that the number of curves in each path decreased after optimization. Moreover, the shape of the path generated by the optimized A* algorithm is much more suitable for the mobile base to track with the local planner which will be introduced later.
**Algorithm 1: **$\Pi^{bm}=\mathrm{HMP}(q_{s},q_{g})$  **Input:** 
$q_{s}:=(q_{s}^{b},q_{s}^{m}),\;q_{g}:=(q_{g}^{b},q_{g}^{m})$  **Output:**  $\mathrm{H-path}:\Pi^{bm}\;\mathrm{from}\;q_{s}\;\mathrm{to}\;q_{g}$
1   $Q:=\mathrm{PLANBASEPATH}(q_{s}^{b},q_{g}^{b})$
2   $\begin{array}{l} \mathrm{Augment}\;\Pi^{\;bm}\;\mathrm{with}\;\mathrm{mobile}\;\mathrm{base}\;\mathrm{path}\;Q,\;\mathrm{and}\;\\ \mathrm{reconfiguration}\;\mathrm{path}\;\pi_{i}^{m}:=\emptyset ,\;\mathrm{specially},\;\pi_{s}^{m}:=q_{s}^{m} \end{array}$
3   if Q≠∅ do
4            
$\mathrm{for}\;\mathrm{all}\;q_{i}^{b}\in Q\;\mathrm{do}$
5                  
$\mathrm{if}\;\mathrm{BASECOLLISIONFREE}(q_{i+1}^{b})\;\mathrm{then}$
6                        
$\mathrm{if}\;q_{i+1}^{b}=q_{g}^{b}\;\mathrm{then}$
7                              
$\mathrm{if}\;\mathrm{MANIPCOLLISIONFREE}(q_{g}^{m})\;\mathrm{then}$
8                                    
$\pi_{i}^{m}:=\mathrm{GENERATEMANIPPATH}(q_{c}^{m})\;$
9                                    
$\pi_{i}^{bm}\leftarrow (q_{i}^{b},\pi_{i}^{m}),\;\pi_{i}^{bm}\in \Pi^{bm}$
10                                     
$\mathrm{MOVEMANIP}(\pi_{i}^{m})$
11                                     
$\mathrm{MOVEBASE}{(q}_{i}^{b}{,q}_{i+1}^{b})$
12                                     
Exit (go to step 32)
13                              
end
14                         
end
15                         
$\left(q_{new}^{m},\pi_{i}^{m}):=\mathrm{PLANMANIPPATH}(q_{c}^{m}\right)$
16                         
$\mathrm{if}\;\pi_{i}^{m}\neq \emptyset \;\mathrm{then}$
17                               
$\mathrm{MOVEMANIP}(\pi_{i}^{m})$
18                               
$\mathrm{MOVEBASE}{(q}_{i}^{b}{,q}_{i+1}^{b})$
19                               
$\pi_{i}^{bm}\leftarrow (q_{i}^{b},\pi_{i}^{m}),\;\pi_{i}^{bm}\in \Pi^{bm}$
20                        
end
21                        
else
22                                    
$Q:=\mathrm{REPLANBASEPATH}(q_{i}^{b},q_{g}^{b},Q)$
23                                    
Continue (go to step 4)
24                        
end
25                
end
26                
else
27                       
$Q:=\mathrm{REPLANBASEPATH}(q_{i}^{b},q_{g}^{b},Q)$
28                       
Continue (go to step 4)
29            
end
30       
end
31 
end
32 
$\mathrm{return}\;\Pi^{bm}$

**Algorithm 2:** $\;Q=\mathrm{PLANBASEPATH}(q_{s}^{b},q_{g}^{b})$         **Input:** 
$q_{s}^{b}:=[x_{s},y_{s},\varphi_{s}],\;q_{g}^{b}:=[x_{g},y_{g},\varphi_{g}]$         **Output:**  $\mathrm{Base}\;\mathrm{path}:Q\;\mathrm{from}\;q_{s}^{b}\;\mathrm{to}\;q_{g}^{b}$
1      
$Q_{origin}:=\mathrm{PLANBASEPATHASTAR}(q_{s}^{b},q_{g}^{b})$
2      
$\mathrm{if}\;Q_{origin}\neq \emptyset \;\mathrm{do}$
3      
$q_{prune}\leftarrow q_{s}^{b}:=[x_{s},y_{s},\theta_{s}]$
4             
$\mathrm{while}\;!q_{prune}=q_{g}^{b}\;\mathrm{do}$
5                  
$q_{target}\leftarrow q_{g}^{b}:=[x_{g},y_{g},\theta_{g}]$
6                  
while pruen+1<target do
7                        
$\mathrm{if}\;\mathrm{COLLISIONFREE}(q_{prune},q_{target})\;\mathrm{then}$
8                              
$\mathrm{Remove}\;q_{j}(prune<j<target)\;\mathrm{from}\;Q_{origin}$
9                              
Break (go to step 15)
10                      
end
11                      
else
12                              
$q_{target}\leftarrow q_{target\text{-}1}$
13                        
end
14                  
end
15                  
$q_{prune}\leftarrow q_{target}$
16            
end
17    
end
18    
$\mathrm{return}\;Q_{origin}$


For the second stage, we augment the full path $\Pi^{bm}$ so that each element $\pi_{i}^{bm}$ has a base pose $q_{i}^{b}$ and a reconfiguration path $\pi_{i}^{m}$. Note that the full path is constructed incrementally as the mobile manipulator moves from start to target.

The second stage described in Lines 3–32 of Algorithm 1 is mainly about local planning. If routine PLANBASEPATH() returns success as shown by the check in Line 3, the mobile base would start moving along the generated path. Otherwise, there is no path available for the mobile base and the motion planning fails. A potential collision checking routine BASECOLLISIONFREE() for the mobile base is used here to check whether the mobile base could move to the next base pose (Line 5). The collision checking for the mobile base can be easily performed using information collected by an onboard sensor such as a lidar. If routine BASECOLLISIONFREE() returns a failure, a replanning routine REPLANBASEPATH()-is called to replan a new *Q* for the mobile base with the updated map, as shown in Algorithm 3. The path from current $q_{i}^{b}$ to target $q_{g}^{b}$ is changed and the mobile base would then follow the new path. If no potential collision is found, the mobile base would try to move to the next base pose. There is a special case, if $q_{i+1}^{b}=q_{g}^{b}$, the mobile base is about to arrive at the target base pose, the manipulator should change its configuration to the target configuration (Lines 6–11). A routine MANIPCOLLISIONFREE() is used here to check whether the manipulator configuration is collision-free with the environment while the mobile base moving straight to the next pose. If routine MANIPCOLLISIONFREE() returns a success, a reconfiguration path $\pi_{i}^{m}$ would be generated by routine GENERATEMANIPPATH(). Here a simple cubic polynomial trajectory is generated. $\Pi^{bm}$ is updated and the motion planning for the mobile manipulator is finally successfully completed. Routine MOVEMANIP() and MOVEBASE() send control signals to the robot. Here, the planner for mobile base is similar to FTC planner [26], where moving straight and rotating are performed in sequence. Here, the mobile base first moves straight to the next position and then rotate to the corresponding direction, which is suitable for the path generated by optimized A*. In most cases, $q_{i+1}^{b}\neq q_{g}^{b}$, routine PLANMANIPPATH() would search for a collision-free configuration and a corresponding reconfiguration path for the manipulator. If routine PLANMANIPPATH() returns a success as shown by the check on Line 16, the mobile manipulator simply moves forward, changing its configuration if necessary, and the $\Pi^{bm}$ is updated. On the other hand, if there is no available configuration or reconfiguration path that could make it feasible for the mobile manipulator to move on, REPLANBASEPATH() is called to replan a new path for the mobile base. This process is repeated until the mobile manipulator arrives at the target or the algorithm fails. 

The pseudocode for PLANMANIPPATH() is shown in Algorithm 4 which is very important for our motion planning method. We will now describe the algorithm in detail. PLANMANIPPATH() routine works as follows: first, it checks whether the current configuration of the manipulator is collision-free with routine MANIPCOLLISIONFREE(). If it is, the safe configuration can be the same as the current configuration and the returned manipulator path is that single configuration. Otherwise, a new safe configuration and corresponding reconfiguration path would be searched (Lines 6–22).
**Algorithm 3:** $Q=\mathrm{REPLANBASEPATH}(q_{ns}^{b},q_{g}^{b},Q)$      
             $q_{ns}^{b}:=[x_{ns},y_{ns},\varphi_{ns}],q_{ns}^{b}\in Q,$         **Input:** 
$q_{g}^{b}:=[x_{g},y_{g},\varphi_{g}],Q:=\{q_{s}^{b},q_{1}^{b},\cdots q_{n}^{b},q_{g}^{b}\}$         **Output:** 
$\mathrm{New}\;\mathrm{base}\;\mathrm{path}:Q\;\mathrm{from}\;q_{s}^{b}\;\mathrm{to}\;q_{g}^{b}$
1      
$Q_{new}=\mathrm{PLANBASEPATH}(q_{ns}^{b},q_{g}^{b})$
2      
$\mathrm{Remove}\;q_{n}(ns<n\leq g)\;\mathrm{from}\;Q$
3      
$\mathrm{Add}\;\mathrm{all}\;q_{j}\in Q_{new}\;\mathrm{to}\;Q$
4      
return Q

**Algorithm 4:** 
$\left(q_{new}^{m},\pi_{i}^{m}):=\mathrm{PLANMANIPPATH}(q_{c}^{m}\right)$
        **Input:** 
$q_{c}^{m}:=[\theta_{1},\theta_{2},\cdots ,\theta_{d}]$
                      
$\mathrm{Collision}-\mathrm{free}\;\mathrm{configuration}:\;q_{new}^{m}$
        **Output:** 
$\mathrm{Reconfiguration}\;\mathrm{path}:\pi_{i}^{m}$
1      
$\mathrm{if}\;\mathrm{MANIPCOLLISIONFREE}(q_{c}^{m})\;\mathrm{then}$
2            
$q_{samp}^{m}\;\leftarrow \;q_{c}^{m}\;$
3            
$\pi_{i}^{m}\;\leftarrow \;q_{c}^{m}\;$
4            
Exit (go to step 24)
5      
end
6      
$q_{temp}^{m}:=\mathrm{MATCHTEMPLATE}()\;$
7      
$q_{samp}^{m}\leftarrow q_{temp}^{m}$
8      
$\pi_{i}^{m}\leftarrow \emptyset \;$
9      
$\mathrm{if}{\;q}_{temp}^{m}\neq \emptyset \;\mathrm{do}$
10            
while !TIMEUP do
11                 
$\mathrm{if}\;\mathrm{MANIPCOLLISIONFREE}(q_{samp}^{m})\;\mathrm{then}$
12                      
$\pi_{i}^{m}:=\mathrm{GENERATEMANIPPATH}(q_{samp}^{m})\;$
13                      
Exit (go to step 24)
14                   
end
15                   
$\mathrm{for}\;\mathrm{all}\;\theta_{i}^{t}\in q_{temp}^{m}\;\mathrm{do}$
16                      
$\mathrm{Sample}\;\theta_{i}^{s}\;\mathrm{around}\;\theta_{i}^{t}\in q_{temp}^{m}$
17                   
end
18                   
$q_{samp}^{m}:=[\theta_{1}^{s},\theta_{2}^{s},\cdots ,\theta_{d}^{s}]$
19              
end
20              
$q_{samp}^{m}\leftarrow \emptyset \;$
21              
$\pi_{i}^{m}\leftarrow \emptyset \;$
22      
end
23      
$\mathrm{return}\;(q_{samp}^{m},\pi_{i}^{m})$


**Figure 3:**
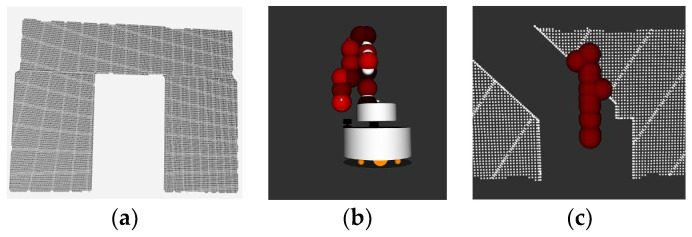
Point clouds and bounding balls for collision checking.

Routine MANIPCOLLISIONFREE() uses point cloud information obtained by the onboard depth camera to check if there is a potential collision between the manipulator and the environment with unchanged configuration as the mobile base moves forward. Different from the 3D collision checking in a 3D map, we use the 2D projection of the manipulator and the 2D projection of the environment in front of the robot to perform the collision checking. Since the mobile base only moves forward in a straight line, this method can detect potential collisions at a small distance and is suitable for door-type scenes. The point cloud obtained from the camera is processed by clipping, filtering, formulating, and then compressed into a plane as shown in Figure 3a. The collision checking is conducted with the point cloud. As for the manipulator, we use bounding balls as shown in Figure 3b. to represent the collision volume of the manipulator, which can be easily projected onto a plane. We select *N* bounding balls for collision checking. For *BALL_n_* (*n* ∈ *N*), the coordinate of the ball center in the *i*th link frame of the manipulator is:

(2) $$C_{bn}^{i}=\left(x_{bn}^{i},\;y_{bn}^{i},\;z_{bn}^{i}\right).$$

For a given configuration $q_{i}^{m}=(\theta_{1},\theta_{2},\theta_{3},\theta_{4},\theta_{5},\theta_{6},\theta_{7})$, the angle of each manipulator joint is known. The coordinate of the center of each bounding ball in the base frame can be calculated:

(3) $$C_{bn}^{0}={\left(x_{bn}^{0},\;y_{bn}^{0},z_{bn}^{0}\right)}^{T}={\;}^{0}T_{i}\cdot {\left(x_{bn}^{i},\;y_{bn}^{i},\;z_{bn}^{i}\right)}^{T}.$$

${\;}^{0}T_{i}$ is the transformation matrix from link *i* to the base.

For each point in the point cloud, the coordinate in the base frame is:

(4) $$P_{k}^{0}=\left(x_{k}^{0},\;y_{k}^{0},\;z_{k}^{0}\right).$$

For the collision checking, only the coordinates on the *y*-axis and *z*-axis are used, the coordinate on the *x*-axis is eliminated. The distance between the point and the center of the bounding ball is:

(5) $$L_{bn,k}^{0}=\sqrt{{\left(y_{bn}^{0}+z_{bn}^{0}\right)}^{2}+{\left(y_{k}^{0}+z_{k}^{0}\right)}^{2}}.$$

The collision checking is shown in Figure 3c. If the distance between the point cloud and the center of the bounding ball is less than the radius of the bounding ball, it is defined that the bounding ball is not in collision with the point. If each bounding ball is not in collision with each point of the point cloud, the manipulator is not in collision with the environment, which means that the current configuration is suitable for the next period of the path.

If a collision is found, the manipulator should change its configuration. Another part of routine PLANMANIPPATH() is the search for a collision-free configuration and the corresponding reconfiguration path (Lines 6–22). The path can be generated by GENERATEMANIPPATH(), which was described earlier, here we proposed a sampling-based heuristic method to find the collision-free configuration. 

**Figure 4:**
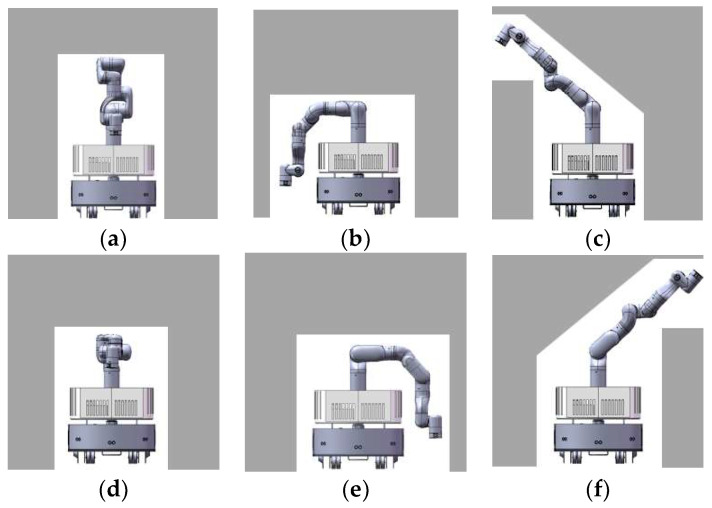
The templates for collision-free configuration. (**a**) normal configuration for the manipulator; (**b**–**f**) template configurations for the manipulator to avoid collision with different types of obstacles.

There are several typical collision-free configurations corresponding to several typical scenes. Looking from the front, we can roughly classify the scene into six types, and we can find the corresponding configuration for the manipulator as shown in Figure 4.

The point cloud used in routine MATCHTEMPLATE() is the same as that used in Routine MANIPCOLLISIONFREE(). The routine analyzes the point cloud and finds the template that most closely matches the point cloud. We divide the whole point cloud into nine regions and count the number of points in each region. The number of points indicates how much of the area is occupied and how likely it is to find a configuration that uses the area. A threshold is chosen for each area, numbers above the threshold mean that it is hard to find a configuration that uses that area without collision and the configuration should avoid that area. According to the complexity of each configuration, each configuration has a priority.

**Figure 5:**
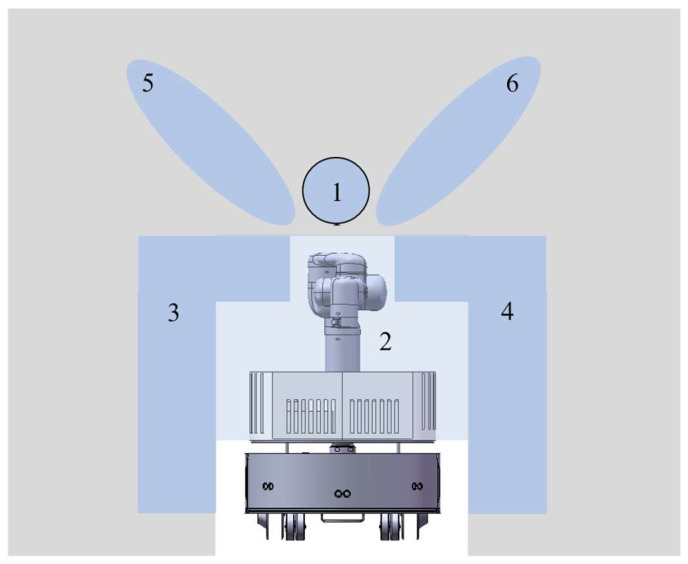
Template matching.

As shown in Figure 5, area 2 must be free otherwise the base of the manipulator cannot change its configuration at all. If this area is occupied, $q_{temp}^{m}=\emptyset$ would be returned. If possible, the configuration using area 2 and 1 should be chosen first, which corresponds to Figure 4a, because it is the normal configuration for the manipulator and has the best stability among all configurations in templates. The situation of area 1 should be checked first. If area 1 is occupied, the situation of area 3 and area 4 is checked at the same time. The configuration corresponding to area 3 and area 4 are shown in Figure 4b,e. The configuration corresponding to areas 5 and 6 are shown in Figure 4c,f, The center of gravity in Figure 4b,e are closer to the center of the mobile base than that in Figure 4c,f. Of course, if area 3 and area 4 are both unavailable, area 5 and area 6 are checked. If both area 5 and area 6 are occupied, the robot has only one choice as shown in Figure 4d. After analyzing all areas, a template that is most similar to the environment would be selected. It is also possible that all areas are occupied, in which case $q_{temp}^{m}=\emptyset$ is returned. After finding the most similar template, all joint angles of the corresponding configuration are known.

**Figure 6:**
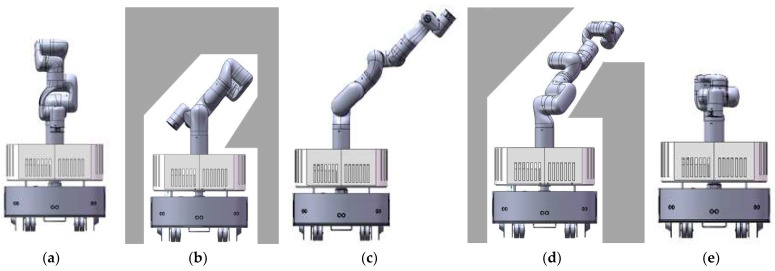
Sampling range and generated configuration. (**a**,**c**,**e**) template configurations for the manipulator; (**b**,**d**) generated configurations with sampling around template configuration (**c**).

A sampling-based method is used to generate the collision-free configuration (Lines 10–19). Each angle is sampled in the selected range near the corresponding angle of the template configuration. The rule of range selection is to make it possible to sample more types of configurations suitable for similar environmental situations. In general, the ranges of the first four angles are relatively more important and should be selected very carefully. As shown in Figure 6a,c,e are template configurations, and b and d are generated configurations with sampling around template Figure 6c. A suitable sampling range of joints could generate a good configuration for variants of the template scene. Meanwhile, selecting a suitable sampling range does not destroy the diversity of configuration, as seen in Figure 6, with the value of the first four joints changing gradually, the template configuration in Figure 6c could change to the template configuration in Figure 6a,e. Thus, if the sampling range is well selected, almost all configurations for passing different shapes of scenes could possibly be generated with one of the templates. This method could improve the success rate of sampling and save sampling time. Then, routine MANIPCOLLISIONFREE() checks whether the sampled configuration is collision free. If it is collision-free, routine GENERATEMANIPPATH() would generate a reconfiguration path and the routine PLANMANIPPATH() would return $\left(q_{samp}^{m},\pi_{i}^{m}\right)$. If a collision is found, another sample is taken, the collision checking is performed, and the process is repeated until a collision-free configuration is found, or the plan time is used up. Our sampling-based heuristic method avoids strange configurations caused by the randomness of the sampling-based method and has better performance with a smaller range of sampling.

## 4. Experiments and Discussion

In this section, we have conducted a series of simulation experiments to evaluate our algorithm. The mobile manipulator in the experiments consists of a differential-drive mobile base and a seven-axis manipulator. A depth camera and a planar lidar are also added to the robot. We import a complete model of the mobile manipulator into ROS [29]. The simulation environment for the experiments is Gazebo which can simulate the real physical properties of the robot and the environment. 

The overall planner is written in C++ and works as a node of ROS. The simulation experiments were carried out under Linux on an Intel Core laptop with 16 GB memory.

**Figure 7:**
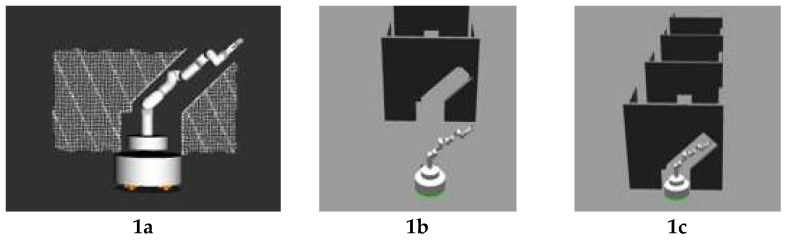

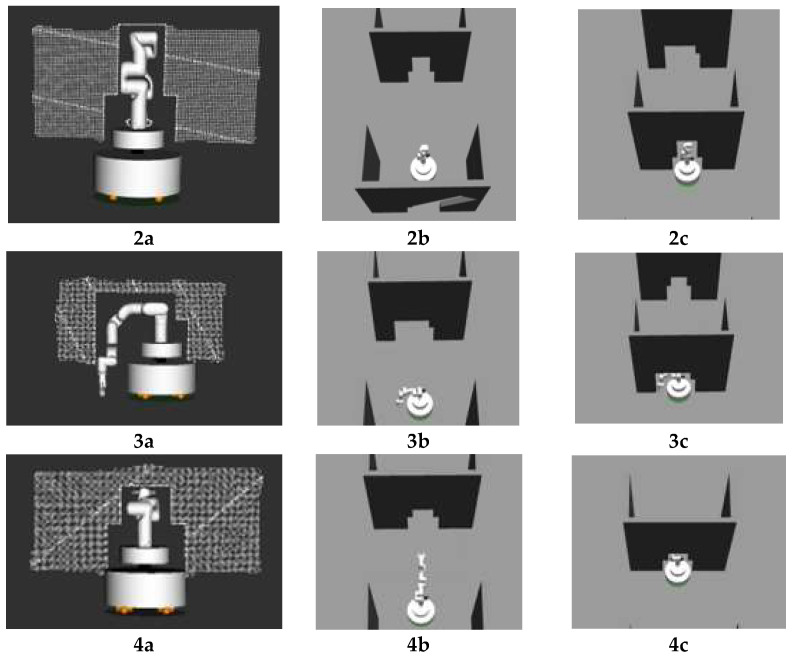
Simulation experiment for sampling-based heuristic method. (**1a**,**2a**,**3a**,**4a**) show the point clouds used in collision checking for scenario 1–4; (**1b**,**2b**,**3b**,**4b**) show the simulation environments of scenario 1–4; (**1c**,**2c**,**3c**,**4c**) show the suitable configurations for the manipulator which would not collide with the environment for scenario 1–4.

At first, we validated the effectiveness of the configuration-generating part of our method which is routine PLANMANIPPATH(), since routine GENERATEMANIPPATH() was simple, we ran sampling-based heuristic method and random sampling method of generating collision-free configuration in four different scenarios in simulation and compared the outcomes. In both methods, routine MANIPCOLLISIONFREE() was used to check whether the configuration is collision-free with the environment. In addition, self-collision checking is added to both methods to avoid possible self-collision of the manipulator. Similar to routine MANIPCOLLISIONFREE(), here bounding balls are used to represent the volume of the manipulator, and the collision checking between bounding balls is performed in 3D with the same method as described in routine MANIPCOLLISIONFREE(). The simulation environment corresponding to each scenario is shown in Figure 7, column (b) while the point cloud used in the collision checking is shown in column (a). The mobile manipulator was placed in front of each obstacle and the configuration-generating algorithm was run. We compared the sampling-base heuristic method with the random sampling method on the basis of three criteria: (a) number of sampling times, (b) sampling time, (c) percentage of successful attempts, and the results are presented in Table 1.

We observe from the table that in all simulation experiments, the random sampling method is outperformed by our sampling-based heuristic method. Our method found the available configuration in less time (with less sampling times) with a much higher success rate compared to the random sampling method. It is worth noting that in sampling-based heuristic method, the reason why the sampled configuration is not available is that the configuration is in collision with the environment. On the contrary, in random sampling method, the reason is always that the configuration is in a self-collision status. It is because in our sampling-based heuristic method, the sampling is taken in a selected range and meanwhile the possible configuration is limited to a range so self-collision rarely occurs. Comparing the results of scenarios 1 and 2 with those of scenarios 3 and 4, when the allowed number of sampling times is changed from 30 to 10, the rate of success of the random sampling method significantly decreases to almost zero while the rate of success of sampling-based heuristic method does not change. It means that the average number of sampling times needed to find the collision-free configuration in the sampling-based heuristic method is much lower than that of the other method, and we can set a lower allowed number of sampling times to save time by avoiding unnecessary sampling attempts in trying to find an available configuration. It can be seen that sampling in the selected range with a template can not only save sampling time with a higher rate of success but also can improve the quality of the generated configuration for that it avoids self-collision situations and self-collision is not needed in the algorithm.

Then, in the same simulation environment shown in Figure 7, we conducted a preliminary experiment to validate the performance of our method in traversing the door-type scenes. Here, the path of the mobile base was given as a straight line while the reconfiguration of the manipulator was tested. As shown in Figure 7, four different obstacles were designed so that the mobile manipulator could pass through them only with a specific collision-free configuration. The mobile manipulator had to pass four obstacles in a row to complete the experiment. As shown in Figure 7 column c, when the mobile manipulator detected an obstacle, it would find the most similar template very quickly and find a suitable configuration for the manipulator which would not collide with the narrow channel. The results are shown in Table 2. The experiment was repeated 10 times and in all experiments the robot was able to change its configuration for each obstacle and reach the goal. The average time of planning for one obstacle is 1.2 s and the average execution time of each experiment is 20.5 s. In this experiment, the linear speed limit of our mobile manipulator was set to 0.5 m/s. Compared to the execution time, the time for path planning is much shorter. 

Next, we could compare our method with a similar planner HAMP [24] and full 9D PRM in a similar scenario in the HAMP experiment [24] where the mobile manipulator was required to navigate from one side of the door to the other side through which the mobile manipulator has to change its configuration. In our experiment, the normal door was changed to a more complex shape as shown in Figure 7(1b). The results are shown in Table 3. In such a scenario, the average time HAMP needed to generate a global plan is 5.7 s, the average time PRM needed is 12.5 s [24], and our method needs 1.1 s on average for all planning including global planning for the mobile base and reconfiguration for the manipulator. With the linear speed limit of our mobile manipulator set to 0.5 m/s, the total execution time of the experiment is 7.3 s, which is just a little longer than the planning time of HAMP and even much shorter than the planned time of full PRM. The scenario in our experiment is more complex but has a more effective performance. However, there are some significant differences between our method and HAMP/full PRM, in HAMP/full PRM, the 3D map of the environment is given, while in our method only the 2D map is given, besides, HAMP/full PRM constructs the global path for both the mobile base and the manipulator at one time before the robot starts moving, while our method constructs the path incrementally as the robot moves. Thus, the comparison here is not so strict due to the differences, but to some extent, it can illustrate the efficiency of our method.

Finally, we conducted an experiment to validate the effectiveness of overall hierarchical motion planning we proposed for mobile manipulators. 

**Figure 8:**
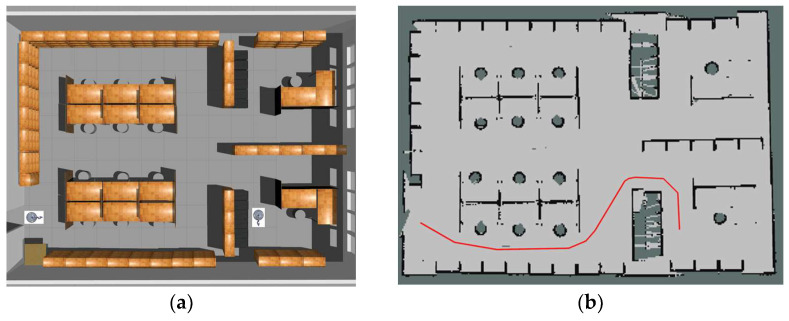
The simulation environment scene.

The simulation scene is shown in Figure 8a. In the simulation environment, we constructed a large room with various obstacles. Before the experiments, we controlled the robot to move around the whole environment and constructed a 2D map. The constructed 2D map is shown in Figure 8b. The start configuration and target configuration of the mobile manipulator are also shown in the figure. The configuration of the start position for the mobile manipulator is (1, 1, 0, 0, 0, 0, 0, 0, 0), and the configuration of the target position is (11, 11, 1.57, 0, 0, 0, 0, 0, 0). The first three parameters are the configuration representations of the mobile base, i.e., the coordinates in the plane and the direction of the mobile base. The last seven parameters are the configuration representations of the manipulator, i.e., the joint angles of the manipulator. The goal of the experiment is to move the mobile manipulator from start to goal configuration without collision with the environment. We added some obstacles in the environment to test whether the motion planning method can deal with the change in the environment.

**Figure 9:**
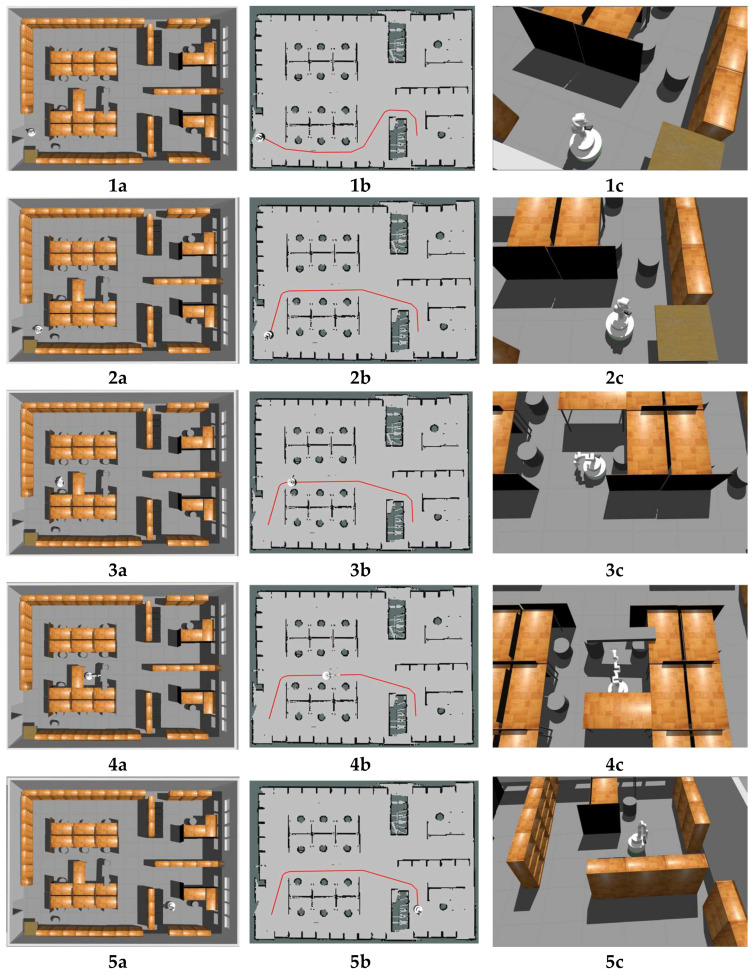
Simulation Experiment. (**1a**,**2a**,**3a**,**4a**,**5a**) show the configurations for the mobile manipulator in the experiment; (**1b**,**2b**,**3b**,**4b**,**5b**) show the global map for the mobile base in the experiment and the red line in each map is the global path; (**1c**,**2c**,**3c**,**4c**,**5c**) show snapshots for the mobile manipulator in the experiment. Please see the text for a description.

The mobile manipulator moved along the path in the start period as if there were no obstacle on the path. The local planner sent control signals to drive the mobile base to precisely follow the path, as shown in the first row of Figure 9. The on-board depth camera and planar lidar collect information about the environment. Collision checking is performed continuously. When the mobile manipulator moved near the round stool, which does not exist in the initial global map, the robot found that there was no way to avoid the obstacle, so a new global path was generated and the robot started to move along the new path, as shown in the second row of Figure 9. Later, the robot moved near a desk which is also not in the initial map, but this time the desk does not block the way of the mobile base, but a possible collision between the manipulator and the desk was detected, the robot had to find a new configuration for the manipulator, according to the shape of the obstacle, a new configuration for the manipulator was found and a path for reconfiguration was generated, then the manipulator changed it configuration and passed the obstacle as shown in the third row of Figure 9. After two obstacles, a new situation occurred, someone had placed a plank between a stool and a desk, leaving only a very narrow space for the robot. Although the mobile base could pass through freely, the manipulator had to find a collision-free configuration. In this situation, a new safe configuration for the manipulator was found, which allowed the whole robot to move through the narrow space, as shown in the fourth row of Figure 9. After these obstacles, the robot finally reached the target configuration, the total time used is 57.8 s on average. 

From the result of the experiment, it can be seen that our hierarchical motion planning method can deal with the change in the environment of its nature of incremental motion planning. Our motion planning method with sampling-based heuristic configuration generating takes less time with a higher success rate than the conservative random sampling method. Compared with those traditional planning algorithms that are purely on the 2D plane, our method could deal with 3D obstacles that could collide with the manipulator. The results showed that it is effective in environments where conservative approaches will fail because the manipulator has to change its configuration several times while navigating from the start to the goal. Compared to those planning algorithms that plan in a global 3D map, our method does not require a 3D representation of the environment, so it avoids large memory requirements and does not need a long period of time to generate a global plan. Both the planning time and the execution time are decreased.

## 5. Conclusions and Future Work

This paper presents a method for mobile manipulator motion planning. The behavioral difference between the mobile base and the manipulator is taken into consideration. Both global planning and local planning are proposed for mobile manipulators. First, the global path for the mobile base is generated with an optimized A* algorithm, and the mobile base would move along the generated path. While the mobile base moves forward to the target configuration, the manipulator may collide with the environment, and the potential collision with the environment is checked. The fast collision checking is performed on a 2D plane perpendicular to the ground and directly in front of the robot. A sampling-based heuristic method is used to find a safe configuration quickly and efficiently for the manipulator. The manipulator would change its configuration several times to avoid collisions with obstacles. Several experiments were conducted in simulation environments. The results show that our planning method can deal with motion planning for mobile manipulators in a 3D environment and have reasonable performance. 

However, our method has some limitations in that it struggles to deal with dynamic and cluttered environments where a collision-free path can hardly be found, and the possible constraints introduced by the manipulation task are not considered in the method. There are several aspects of our method that could be improved in future work. First, with the development of robotic object perception [30,31,32], the mobile manipulator could build a small-scale representation of the environment around itself with more object information, which would be very helpful to deal with complex 3D environments and have better manipulation capabilities. In addition, the manipulation task introduces many constraints to the motion planning, therefore, the whole-body motion planning method including redundancy resolution [33] and base position optimizition [34] has the potential to be added to our proposed method to plan a path satisfying various constraints introduced by the environment and the manipulation task. Furthermore, the real-time whole-body planning method is another issue that deserves further attention.

## Figures and Tables

**Table 1:** Experimental results in 4 scenarios for reconfiguration.

Scenario ID	Method	Permitted # of Sampling Times	# of Self-Collision Times Mean	# of Collision with Environment Times Mean	# of Sampling Times Mean	Sampling Time(s) Mean	#Succ. /#Runs
1	Our method Random Sampling	30	0	6.4	7.3	0.6	28/30
			22.3	27.5	27.7	2.6	7/30
2	Our method Random Sampling	30	0	2.1	3.1	0.3	30/30
			20.5	26.1	26.3	2.7	5/30
3	Our method Random Sampling	10	0	1.7	2.7	0.2	29/30
			7.3	9.8	9.8	0.9	1/30
4	Our method Random Sampling	10	0	1.3	2.3	0.2	30/30
			7.7	10	10	0.9	0/30

**Table 2:** Experimental results in passing four doors in a row.

Method	Plan Time(s) Mean	Run Time(s)	#Succ./#Runs
HMP	1.2	20.5	10/10

**Table 3:** Experimental results in passing a low door (hmp vs. Hamp vs. Full prm).

Method	Plan Time(s)	Run Time(s)
HMP	1.1	7.3
HAMP	5.7	N/A
Full PRM	12.5	N/A

## Data Availability

Not applicable.
